# Healing with Love: Oxytocin Accelerates Oral Ulcer Recovery by Reducing Inflammation

**DOI:** 10.3390/jcm14082667

**Published:** 2025-04-14

**Authors:** Mert Zeytinoğlu, Osman Sezer Çınaroğlu, Ejder Saylav Bora, Oytun Erbaş

**Affiliations:** 1 Department of Oral, Dental and Maxillofacial Surgery, Faculty of Dentistry, Ege University, 35040 Bornova, Izmir, Türkiye; mert.zeytinoglu@ege.edu.tr; 2 Department of Emergency Medicine, Faculty of Medicine, Izmir Katip Çelebi University, 35620 Çiğli, Izmir, Türkiye; drsezer@hotmail.com; 3 Faculty of Medicine, BAMER, Biruni University, 34015 Zeytinburnu, Istanbul, Türkiye; oytunerbas2012@gmail.com

**Keywords:** oral ulcerative mucositis, oxytocin, MMP-2/TIMP-2 pathway, inflammation, oxidative stress

## Abstract

**Background:** Oral ulcerative mucositis (OUM) is a painful, inflammatory mucosa lesion that impairs quality of life. Despite available treatments, effective agents that promote faster healing and modulate inflammation are still needed. Oxytocin (OT), a neuropeptide with anti-inflammatory and antioxidant properties, may aid wound healing by regulating the remodeling of the extracellular matrix (ECM). This study investigates the effects of OT on oral ulcer healing in rats, focusing on its modulation of the MMP-2/TIMP-2 pathway. **Methods:** Acetic acid 70% was used as the oral mucosal ulcer inducer. Thirty-six Wistar albino rats were divided into control, oral ulcer + saline, and oral ulcer + OT (intraperitoneally for 15 days) groups. Histopathological, biochemical, and molecular analyses were performed. Buccal mucosa tissue was examined for TNF-α, TIMP-2, and MMP-2 levels via ELISA, while oxidative stress markers and pentraxin-3 (PTX3) were also assessed. **Results:** OT significantly preserved epithelial integrity and reduced fibrosis compared to the saline group (*p* < 0.001). TNF-α, MMP-2, PTX3, and malondialdehyde levels were significantly lower, while TIMP-2 levels were elevated in the OT-treated group (*p* < 0.01). Histopathological analysis confirmed reduced inflammation and enhanced tissue organization. **Conclusions:** OT accelerates oral ulcer healing by modulating inflammation, oxidative stress, and ECM remodeling via the MMP-2/TIMP-2 pathway. These findings highlight its potential as a therapeutic agent for managing mucosal injuries. Further clinical studies are warranted.

## 1. Introduction

Oral ulcerative mucositis (OUM) is a prevalent acute hazard of antineoplastics. It is characterized by an inflammatory reaction that can impact oncologic treatment patients [1]. The incidence varies from 15% to 100%, contingent upon the dosage of chemotherapeutic (CT) and neck and head radiotherapy (RT) [2].

A complex interaction of factors, including inflammatory and oxidative damage, predominantly influences OUM. Research indicates that chemotherapy agents such as 5-fluorouracil (5-FU) and irinotecan can cause considerable mucosal damage, marked by ulceration, inflammation, and the disruption of mucosal architecture [3]. These agents elevate the expression of cytokines that cause inflammation and oxidative stress indicators, including NF-κB and the production of reactive oxygen species (ROS), which play a role in the pathogenesis of OUM [4,5].

Diverse therapeutic modalities for oral mucositis have been employed, including cryotherapy, laser therapy, and creating a radiation shield to safeguard oral tissues during irradiation. Various mouthwash options are utilized due to their anti-inflammatory, anesthetic, antipyretic, analgesic, and antimicrobial properties [6]. Moreover, systemically administered pharmacological agents, including pentoxifylline, thalidomide, and simvastatin, correlate with the onset and severity of all problems reported in transplantation patients [6]. Clinical trials have indicated that these medications diminish the incidence and intensity of significant complications, including oral mucositis. Notwithstanding these treatment options, additional cost-effective methods to avert mucositis remain necessary [7].

Oxytocin (OT) is a cyclical nonapeptide produced in the cell bodies of the paraventricular nuclei of the brain’s hypothalamus and conveyed via the axons of those cells to the posterior pituitary [8]. It is well-known for its functions in relationships, reproduction, and numerous physiological processes, including the repair of tissues [9]. Besides its traditional functions, OT exhibits antioxidant, anti-anxiety, and anti-inflammatory properties across various metabolic pathways [10]. Recent research has investigated its potential to improve wound healing and tissue regeneration, providing intriguing insights into its therapeutic uses [9,11].

Tissue inhibitors of metalloproteinases (TIMPs) play a vital role in modulating matrix metallo-proteinases (MMPs), which are implicated in tissue remodeling and cancer advancement. TIMP-2 significantly modulates the function of MMP-2, a protease involved in the disintegration of extracellular matrix elements. Comprehending the expression levels of TIMP-2 in the buccal mucosa, particularly regarding oral diseases, is crucial for clarifying its function in disease progression and its potential as a therapeutic target. The disparity in TIMP-2 and MMP-2 expression among healthy oral tissue and dysplasia was significantly different, indicating that TIMP-2 may be significant in advancing premalignant lesions [12]. The expression of TIMP-2 and MMP-2 was not detected in healthy oral tissue [13]. It is known that pentraxin-3 can be used as an indicator of inflammation that may be effective in predicting clinical outcomes in periodontal diseases as well as cardiovascular diseases, chronic kidney disease, and rheumatologic diseases [14].

OUM is very frequent, painful, and impairs quality of life. In this study, we aim to investigate the histological and biochemical changes after OT admission in the OUM model in rats.

## 2. Materials and Methods

### 2.1. Animals

This study utilized 36 male Wistar albino adult rats weighing 200 and 250 g. The experiments conducted in this study adhered to the guidelines outlined in the Guide for the Care and Use of Laboratory Animals established by the National Institutes of Health (USA) [15]. They obtained approval from the Animal Ethics Committee (Science University, Ethical number: 1325012308). The rats utilized in the experiment were procured from the Experimental Animal Laboratory of Science University. Rats were fed ad libitum and kept in pairs within steel cages in a temperature-controlled setting (22 ± 2 °C) with 12 h light/dark cycles. Animals were matched for age and weight to reduce biological variability. Environmental factors such as housing conditions, diet, and circadian light cycles were kept constant across groups.

### 2.2. Experimental Protocol

A model of oral ulceration [16,17,18] was established by the left buccal injection of a 70% acetic acid solution (AA) (Merk Sigma-Aldrich, Burlington, MA, USA) to 24 rats. No pharmacological intervention was provided to the remaining rats (n = 12) (normal control group).

Following ketamine anesthesia (50 mg/kg), 70% acetic acid was applied for 60 s to the dry oral mucosa using a regular-sized cotton tip applicator (5.0 mm) saturated in 70% acetic acid for 3−5 s [18].

Subsequently, 24 rats with oral ulcer models were randomly assigned to two groups. Group 1 rats (saline group, 12 rats) received 1 mL/kg/day of 0.9% NaCl (saline) by intraperitoneal injection. In contrast, group 2 rats (OT group, 12 rats) were administered 0.1 mg/kg/day of OT via the same route.

All treatments were administered for 15 days. After the trial, all animals were euthanized via cervical dislocation under anesthesia (Ketamine 100 mg/kg, Richterpharma AG Austria, and xylazine 50 mg/kg, Bayer, Germany) and blood samples were obtained through heart puncture for biochemical examination. The left buccal mucosa, measuring 10 mm, was excised for histological and biochemical analysis. To mitigate potential methodological biases, we have taken several steps: the random allocation of animals to study groups, use of blinded histopathological assessment, and replication of ELISA and biochemical measurements in duplicates.

### 2.3. Histopathological Evaluation of Buccal Mucosa

Formalin-fixed buccal slices (4 μm) underwent staining with hematoxylin and eosin. All sections were captured using an Olympus C-5050 (Olympus Corp., Tokyo, Japan) digital camera affixed to an Olympus BX51 microscope (Olympus Corp., Tokyo, Japan).

Scores were allocated based on the subsequent criteria: The parameters of the epithelium and fibrosis were assessed. Epithelium characteristics were compared to normal mucosa and represented as percentage values. Fibrosis parameters were represented as percentage values [19].

### 2.4. Evaluation of Plasma Pentraxin-3 Levels

Plasma pentraxin-3 (PTX3) concentrations were quantified in each 100 μL sample using standard ELISA equipment at 450 nm using a PTX3 kit (Uscn Life Science Inc., Wuhan, China). PTX3 levels were assessed in duplicate following the manufacturer’s instructions.

### 2.5. Buccal Mucosa Biochemical Analysis for TNF-α, TIMP-2, and MMP-2

Following decapitation, the buccal mucosa was promptly excised and preserved at −20 °C for further biochemical investigation. Entire buccal mucosa tissues have been homogenized using a glass homogenizer in 5 volumes of phosphate-buffered saline (pH 7.4) and subsequently centrifuged at 5000× *g* for 15 min for tissue analysis. The supernatant was subsequently collected, and the total protein content in the liver homogenates was quantified using Bradford’s technique using the albumin from bovines as the standard [20].

TNFα, TIMP-2, and MMP-2 concentrations in the buccal mucosa supernatants were quantified using publicly available rat enzyme-linked immunosorbent assay (ELISA) kits. According to the manufacturer’s instructions, each specimen from each animal was assessed in duplicate. A microplate reader (MultiscanGo, Thermo Fisher Scientific Laboratory Equipment, NH, USA) was employed to measure absorbance levels.

### 2.6. Measurement of Plasma Lipid Peroxidation

Lipid peroxidation in plasma samples was assessed by quantifying malondialdehyde (MDA) levels [21] as substances that react with thiobarbituric acid (TBARS). Trichloroacetic acid and TBARS reagent were introduced to the plasma samples, subsequently combined, and incubated at 100 °C for 60 min. After ice chilling, the samples were spun at 3000 rpm for 20 min, and the absorbance of the supernatant was measured at 535 nm. MDA concentrations were quantified in nanomoles per liter (nM), utilizing tetraethoxypropane for calibration [21].

### 2.7. Statistical Analysis

Data analysis was conducted utilizing SPSS version 15.0 for Windows. The parametric variable groups were compared utilizing the Student’s *t*-test and analysis of variance (ANOVA). The Mann–Whitney U test was employed to compare the groups of nonparametric variables. The results were presented as mean ± standard error of the mean (SEM). A *p*-value of less than 0.05 was deemed statistically significant. *p* < 0.001 was deemed statistically very significant.

## 3. Results

The epithelial score indicated that the epithelial integrity was fully maintained in the control group. The saline-treated group exhibited a marked deterioration in epithelial integrity, with the epithelial score declining to 32.4 ± 3.9 (*p* < 0.001) (Table 1). The OT-treated group exhibited the superior preservation of epithelial integrity compared to the saline group, with a score of 65.1 ± 5.5 (*p* < 0.001). Minimal fibrosis was noted in the control group (4.0), but the fibrosis score escalated to 78.2 ± 4.3 in the saline group (*p* < 0.01). In the OT group, the fibrosis score was 24.3 ± 7.7, showing a significant decrease compared to the saline group (*p* < 0.001). Instead of reporting statistical significance only by the *p*-value, effect sizes of intergroup differences were also calculated and interpreted. Especially in epithelial integrity scores, the Cohen’s d value between Saline and Oxytocin groups was calculated as ≈ 1.93 (M_1_ = 32.4, SD_1_ = 3.9; M_2_ = 65.1, SD_2_ = 5.5). This value represents a very large effect. Similarly, Cohen’s d ≈ 2.48, calculated in terms of fibrosis scores, indicating a high strength of treatment effect.

In the control group, the epithelial integrity was fully maintained. The saline-treated group exhibited a significant impairment in epithelial integrity, with the epithelial score decreasing to 32.4 ± 3.9 (*p* < 0.001). The OT-treated group exhibited the superior preservation of epithelial integrity compared to the saline group, with a score of 65.1 ± 5.5 (*p* < 0.001). In terms of fibrosis, minimal fibrosis was observed in the control group (4.0), while the fibrosis score increased to 78.2 ± 4.3 in the saline group (*p* < 0.01). The fibrosis score in the OT group was 24.3 ± 7.7, indicating a significant reduction relative to the saline group (*p* < 0.001) (Table 1).

The plasma biochemical parameters indicated that the level of pentraxin-3, an inflammation marker, rose from 0.7 ± 0.1 in the control group to 1.87 ± 0.2 in the saline group (*p* < 0.01). In the group treated with OT, the measured level was 1.09 ± 0.1, indicating a significant reduction in inflammation (*p* < 0.01). The malondialdehyde levels, indicative of oxidative stress, were measured at 47.4 ± 3.9 in the control group and 73.6 ± 2.4 in the saline group (*p* < 0.01). The value in the OT-treated group decreased to 50.2 ± 1.8 (*p* < 0.01) (Table 1).

The concentration of TNF-α, an inflammatory marker in the buccal mucosa, rose from 68.6 ± 2.5 in the control group to 103.05 ± 8.2 in the saline group (*p* < 0.01). The OT-treated group exhibited a TNF-α level of 92.8 ± 4.2, significantly reducing inflammation (*p* < 0.01). The TIMP-2 level, indicative of tissue regeneration, was measured at 211.5 ± 10.8 in the control group, whereas it decreased to 39.9 ± 3.3 in the saline-treated group (*p* < 0.001). The OT-treated group exhibited an increase in TIMP-2 levels to 116.8 ± 6.5, which was found to contribute to tissue repair (*p* < 0.001). The MMP-2 level linked to tissue destruction was recorded at 5.19 ± 0.2 in the control group, while it rose to 11.2 ± 1.2 in the saline-treated group (*p* < 0.01). In the group treated with OT, the MMP-2 level measured 4.5 ± 0.8, and a significant reduction in tissue destruction was noted (*p* < 0.01) (Table 1).

**Figure 1:**
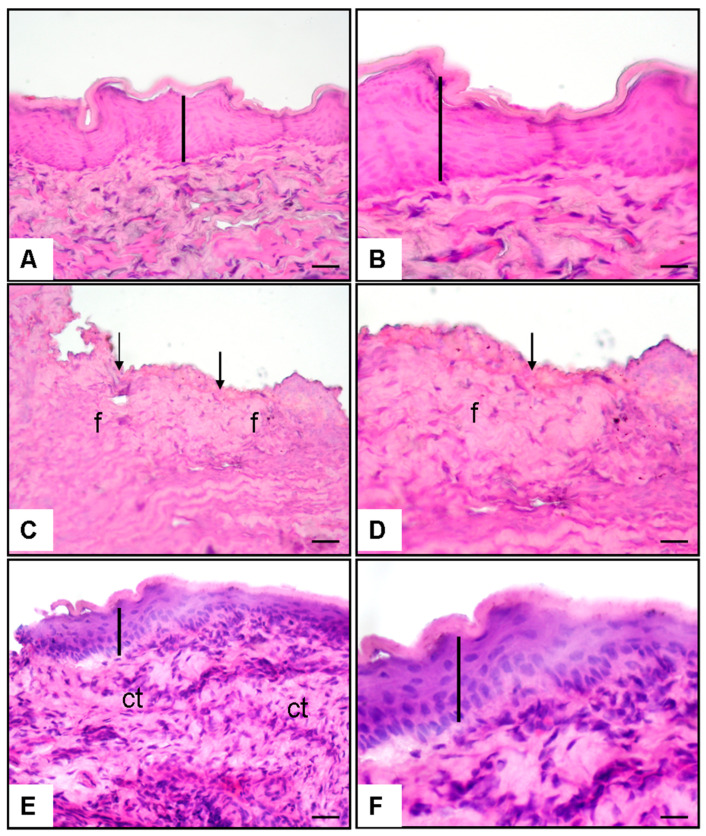
Histopathological findings × 20 and 40 magnification, hematoxylin, and eosine stain. (**A**,**B**) Normal control group buccal mucosa, normal epithelium (line); (**C**,**D**) oral ulcer and % 0.9 NaCl given group buccal mucosa, disrupted epithelium (arrow), and fibrosis (f); (**E**,**F**) oral ulcer and OT given group buccal mucosa, healed epithelium (line), and connective tissue (ct).

The histological assessment revealed that the epithelial integrity was fully maintained, and the connective tissue exhibited a homogenous structure in the buccal mucosa sections of the control group. The epithelial layer was found to possess a regular structure and was connected with the underlying connective tissue. In the group where mouth ulcers were produced and treated with 0.9% NaCl, epithelial integrity was considerably compromised, surface abnormalities emerged, and epithelial loss transpired. In this cohort, regions of fibrosis expanded, and a pronounced inflammatory response was noted in the connective tissue (Figure 1).

In the OT-treated group, the epithelial integrity was predominantly maintained; the epithelial surface had a more uniform structure, and the underlying connective tissue was superior to the NaCl group. OT administration resulted in a significant decrease in the fibrosis rate, an alleviation of inflammation, and a more balanced restructuring of connective tissue (Figure 1).

## 4. Discussion

The management of OUM is problematic due to the dynamic and moist conditions of the oral cavity, which may impede the efficacy of topical therapies [22]. In this context, there is still no common consensus on the treatment of OUM.

Many herbal, local, and systemic treatments have been tried to prevent and treat OUM. For example, Sauted garden burnet root, Baikal skullcap root, cutch, borneol, and borax have effectively treated oral ulcers without toxic side effects. This formulation is noted for its quick healing properties [23]. In an experimental study, coconut milk demonstrated superior healing effects on oral ulcers compared to a control group [18]. Herbal medicines are safer than synthetic drugs for treating oral ulcers, providing symptomatic relief without severe adverse effects [24]. All of them are recommended as a natural, safe, and inexpensive alternative for ulcer treatment. Hydroxypropyl cellulose-based Orally Dissolving Films (ODFs) infused with dexamethasone have demonstrated potential in treating oral ulcers. These films provide swift action onset and facilitate administration, improving drug solubility and dissolution rates [25]. Furthermore, Betamethasone-embedded dissolvable microneedle patches offer a non-invasive and efficient means of administering medication directly to the ulcer location, facilitating healing and diminishing inflammation [26]. Consequently, both implications have been formulated to modulate inflammatory pathways and enhance antibacterial properties to expedite healing. Similarly, in this study, the anti-inflammatory and also oxidative damage protective effect of OT was proven by biochemical evaluations as the suppression of pentraxin-3, which is an indicator of the inflammatory process, and suppression of MDA, which is an indicator of oxidative damage in this context; the results are in parallel with local applications. Moreover, Iseri et al. reported that OT decreased the expression of proinflammatory cytokines such as TNF-α, thus attenuating the severity of inflammation as marked in this study [11].

As mentioned, drugs used locally for OUM have a low level of evidence, and their use has not become widespread. Systemic drugs used in the case of oral ulcers or to prevent oral ulcers from occurring have been studied in diseases such as Behcet’s syndrome, Crohn’s disease, HIV infection, or Reiter’s syndrome [27,28]. For recurrent oral ulcers, the currently used drugs are Colchicine, Rebamipide, and Azathioprine. Colchicine may treat various diseases by influencing the expression and activation of proteins like MMP-2 and TIMP-2 [29]. For instance, Colchicine increases the degree of fibrosis in rats with liver fibrosis by reducing MMP-2 expression, which relates to Colchicine’s role in inhibiting MMP-2 expression as part of its anti-fibrotic effects [30,31]. Similarly, OT in this study inhibits MMP-2 expression and augments MMP-2 levels. These results suggest that OT provokes wound healing in the buccal mucosa through a potent modulatory effect of TIMP-2 without creating fibrotic tissue.

A study by Dymetrenko et al. claims that OT significantly diminishes gingival inflammation and bone resorption in a laboratory model of ligature-induced periodontal disease in adult male rats [32]. Moreover, Xu et al. found that OT enhances the survival rate of tissue flaps. This effect is linked to OT’s enhancement of microcirculation and angiogenesis, leading to improved blood flow to the tissue [33]. Consistent with our study’s findings, El-Sherbiny et al. [34] noted that OT may facilitate the repair of diverse tissue injuries by mitigating inflammatory responses and oxidative stress, suggesting its potential as a therapeutic agent. OT also mitigates the adverse effects of stress on wound healing. Environmental enrichment and OT administration have demonstrated efficacy in enhancing healing rates in stressed animals, indicating their role in modulating stress responses [35].

Similar to the study of Sorg et al. [36], histopathological analysis revealed that OT administration significantly suppressed inflammation and fibrosis development severity by preserving epithelial integrity. These findings strongly suggest that the inflammatory response after an oral ulcer may negatively affect the healing process by accelerating tissue destruction. In the OT-treated group, epithelial integrity was mainly preserved, the surface structure maintained regularity, and the connective tissue organization was significantly improved.

OT controls the degradation of ECM by regulating the MMP/TIMP balance, which may allow the tissue repair process to proceed more physiologically and balanced. This mechanism points to a wide range of effects that may not only be limited to the suppression of inflammation, but also accelerate wound healing by promoting tissue regeneration. Studies in the literature on the antifibrotic and tissue-protective properties of OT largely overlap with the findings obtained and confirm the potential of OT to regulate tissue homeostasis [37,38].

The decrease in TIMP-2 levels, which support tissue regeneration, may accelerate the tissue destruction process and the disruption of ECM homeostasis with the triggering of the inflammatory response after an oral ulcer. The increase in TIMP-2 levels with OT administration indicates that this hormone may support tissue regeneration and prevent matrix degradation. OT may accelerate tissue repair by regulating the balance between MMPs and tissue inhibitors (TIMPs), thus protecting the epithelial integrity and positively affecting wound healing.

Social interactions can diminish stress hormones by enhancing OT secretion, thereby expediting wound healing. Social isolation adversely impacts wound healing; however, OT administration can mitigate these effects [39,40].

Regenerative dentistry is an emerging field in our world, but still remains experimental. Bone Morphogenetic Protein 7 (BMP7) [41], Platelet-Rich Plasma, Platelet-Rich Fibrin [42], human dental pulp stem cells [43], Calcium Phosphate and Silicate-Based Materials [43], epigenetic therapies such as histone deacetylase inhibitors [44], and the combination of biological agents with bone grafts [45] are being tested on humans, but their long-term effects are unknown, and they are still costly methods. Biological agents have proven effective in preventing alveolar bone loss and mitigating tissue inflammation during the progression of experimental periodontitis. These agents markedly curtailed bone loss and diminished proinflammatory mediators [46]. Moreover, naturally occurring biological agents, such as algae, exhibit potential in the management of periodontitis due to their anti-inflammatory and antioxidant characteristics [47].

OT has been found to increase bone regeneration [48], the differentiation of dental pulp stem cells [49], periodontal ligament stem cells [50], and the expression of osteogenic and odontogenic marker genes [51]. However, no studies on local or systemic administration to oral cavity tissue are available yet. This situation emphasizes the need for further studies examining the possible effects of OT on tissue regeneration and matrix dynamics in more detail.

### Limitations

Limitations of this study include the relatively small sample size, the use of an animal model that may not fully represent human physiology, the lack of long-term follow-up to assess sustained effects or relapse, and the fact that only peripheral, not central, effects of OT were evaluated.

## 5. Conclusions

The results of this study support the use of OT in oral ulcerous lesions alongside other systemic drugs. OT significantly affects preserving epithelial integrity, reducing inflammation, promoting tissue regeneration, and minimizing oxidative stress after oral ulcers. However, the mechanisms of action of OT need to be elucidated in more detail, and comprehensive studies should be carried out on its clinical applications.

## Figures and Tables

**Table 1:** Biochemical results and histopathological results were presented as mean ± SEM. Statistical analyses were performed using one-way ANOVA. * *p* < 0.01, ** *p* < 0.001 different from normal groups; # *p* < 0.01, ## *p* < 0.001 different from oral ulcer and saline group.

	Control Group	Oral Ulcer and % 0.9 NaCl Group	Oral Ulcer and OT Group
Histopathological score Epithelium	100	32.4 ± 3.9 **	65.1 ± 5.5 ##
Fibrosis	4.0	78.2 ± 4.3 *	24.3 ± 7.7 ##
Plasma Pentraxin-3 level (ng/mL)	0.7 ± 0.1	1.87 ± 0.2 *	1.09 ± 0.1 #
Plasma MDA level (nM)	47.4 ± 3.9	73.6 ± 2.4 *	50.2 ± 1.8 #
Buccal Mucosa TNF-alfa level (pg/g tissue)	68.6 ± 2.5	103.05 ± 8.2 *	92.8 ± 4.2 #
Buccal Mucosa TIMP-2 level (pg/g tissue)	211.5 ± 10.8	39.9 ± 3.3 **	116.8 ± 6.5 ##
Buccal Mucosa MMP-2 level (pg/mg tissue)	5.19 ± 0.2	11.2 ± 1.2 *	4.5 ± 0.8 #

## Data Availability

Data are available upon reasonable request from the corresponding author.
